# The transcriptional landscape of a hepatoma cell line grown on scaffolds of extracellular matrix proteins

**DOI:** 10.1186/s12864-021-07532-2

**Published:** 2021-04-06

**Authors:** Souvik Ghosh, Anastasiya Börsch, Shreemoyee Ghosh, Mihaela Zavolan

**Affiliations:** 1 Biozentrum, University of Basel, Basel, Switzerland; 2 Swiss Institute of Bioinformatics, Lausanne, Switzerland

## Abstract

**Background:**

The behavior of cells in vivo is complex and highly dynamic, as it results from an interplay between intercellular matrix proteins with surface receptors and other microenvironmental cues. Although the effects of the cellular niche have been investigated for a number of cell types using different molecular approaches, comprehensive assessments of how the global transcriptome responds to 3D scaffolds composed of various extracellular matrix (ECM) constituents at different concentrations are still lacking.

**Results:**

In this study, we explored the effects of two diverse extracellular matrix (ECM) components, Collagen I and Matrigel, on the transcriptional profile of cells in a cell culture system. Culturing Huh-7 cells on traditional cell culture plates (Control) or on the ECM components at different concentrations to modulate microenvironment properties, we have generated transcriptomics data that may be further explored to understand the differentiation and growth potential of this cell type for the development of 3D cultures. Our analysis infers transcription factors that are most responsible for the transcriptome response to the extracellular cues.

**Conclusion:**

Our data indicates that the Collagen I substrate induces a robust transcriptional response in the Huh-7 cells, distinct from that induced by Matrigel. Enhanced hepatocyte markers (ALB and miR-122) reveal a potentially robust remodelling towards primary hepatocytes. Our results aid in defining the appropriate culture and transcription pathways while using hepatoma cell lines. As systems mimicking the in vivo structure and function of liver cells are still being developed, our study could potentially circumvent bottlenecks of limited availability of primary hepatocytes for preclinical studies of drug targets.

**Supplementary Information:**

The online version contains supplementary material available at 10.1186/s12864-021-07532-2.

## Background

The liver is a critical hub for numerous physiological processes. These include macronutrient metabolism, blood volume regulation, immune system support, endocrine control of growth signaling pathways, lipid and cholesterol homeostasis, and the breakdown of xenobiotic compounds, including many current drugs [1]. It is composed of about 80% hepatocytes and 20% non-parenchymal cells such as stellate, sinusoidal endothelial, and Kupffer cells [1]. Isolated hepatocytes cultured on surfaces (2D) in polystyrene-coated culture flasks lose their native morphology, polarity and functionality, which subsequently limits their effectiveness in applications such as toxicity screening of drug metabolites [2]. However, it has been reported that culturing primary hepatocytes between Collagen layers could be a better alternative [3, 4], as these three-dimensional (3D) cultures proved to be better in maintaining hepatocyte phenotype and cell polarization.

The ECM (Extracellular Matrix) is a dynamic structure that provides a supportive scaffold and actively regulates biological functions of cells, at least partly through interactions of ECM components with specific cell surface molecules [5]. The 3D matrices that are used to study cell behavior in a tissue-like environment in normal and pathological conditions are often made primarily of Collagen, amongst all other components of the ECM [6, 7].

The development of a culture system mimicking the in vivo structure and functions of liver cells still remains a challenge. We sought to contribute to resolving this challenge by molecular characterization of a cell line-based experimental system that exhibits hepatocyte-like polarity. The system is based on Huh-7, a well-established, differentiated hepatocyte cell line that was generated in 1982, from a liver carcinoma of a 57-year-old Japanese male [8]. The ability to propagate Huh-7 cells in media containing defined components broadens the relevance of this cell line, from a model of oncogenesis, to one which is also suitable for the elucidation of regulatory mechanisms of gene expression. The properties of these hepatoma cells allow systematic studies of in vitro effects of various compounds on their growth and metabolism [9]. An additional application of the Huh-7 cell line is based on its permissiveness to Hepatitis C virus genomic replication.

Both pathophysiological changes as well as the regeneration of the liver are accompanied by the remodelling of the ECM [10–12]. To gain insight into the molecular changes that contribute to the phenotypes observed for different culture conditions, we explored the 3D growth of Huh-7 cells on different concentrations of Collagen I matrix. Furthermore, in line with previous studies, we also used Matrigel in several concentrations, as another basement membrane matrix mimic. Matrigel is a gelatinous protein mixture secreted by the Engelbreth–Holm–Swarm mouse sarcoma cells that resembles the complex extracellular environment found in many tissues and is used to produce thick 3D gels for cell culture [13]. It has previously also been documented that Matrigel-cultured Huh-7 cells assembled into 3D spheroid like structures (henceforth referred to as spheroids), whereas standard 2D-cultured cells formed the typical epithelial monolayer [14]. The cues provided by the niche are known to be important for modulating cellular behaviors such as differentiation, viability, and proliferation. Being able to modulate these behaviors via simple changes in the concentration of matrix components would be very convenient for smart scaffold designs.

In the current study, we identify several transcription factors that modulate the gene expression profile of the cells cultured in different conditions. Furthermore, we also observe that the activity of several of these factors changes linearly with changes in concentration of matrices used for growth which implies robust roles of these factors and their involved pathways. Interestingly, the analysis of the data set also reveals the great heterogeneity in the incurred changes between the two different types of matrices used to seed growth. Our data also reveals overall changes in critical hepatic functions, like insulin response, drug metabolism, and amino acid catabolism, highlighting the roles of extracellular matrix components on liver function. We propose that this comprehensive analysis of the gene expression profiles and the corresponding transcription factor activities will be informative for the design of matrix scaffolds that support the 3D growth of cells and the formation of liver organoids.

## Results

**Fig. 1:**
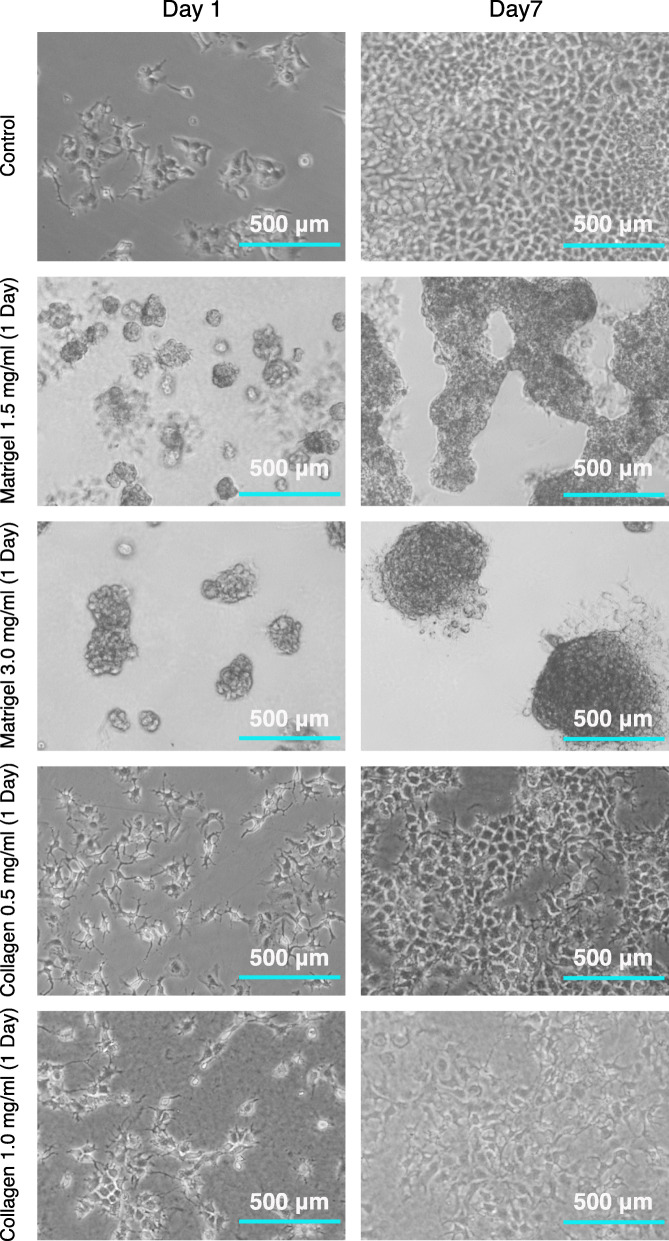
Microscopic analysis of growth of Huh-7 cells on Collagen and Matrigel. Phase contrast images of Huh-7 cells grown on the indicated extracellular membrane matrices or Control culture medium at day 1 and day 7 post seeding were captured at 10X objective zoom levels. The indicated scale bar represents 500 μm

To validate the morphological phenotypes reported earlier for the growth of Huh-7 cells on Matrigel basement membrane matrices [14] and compare them to those observed on Collagen matrices of different concentrations, we imaged the growing cells at day 1 and day 7 after seeding as mentioned in the methods section (Fig. 1). Huh-7 cells grown on basement membrane matrices demonstrated a strikingly different morphology in comparison to cells grown on Control, polystyrene coated cell culture plates (Fig. 1). In addition, cells cultured on Matrigel gave rise to distinct spheroids, which was not the case for cells that are cultured on Collagen. This is in line with what has been described before [15], where it was further observed that the spheroids formed in Matrigel expressed sinusoid markers, whereas the cells that were cultured in Collagen did not. Nevertheless, the Collagen-cultured cells were found to stabilize their synthetic and enzymatic activity a week after isolation.

**Fig. 2:**
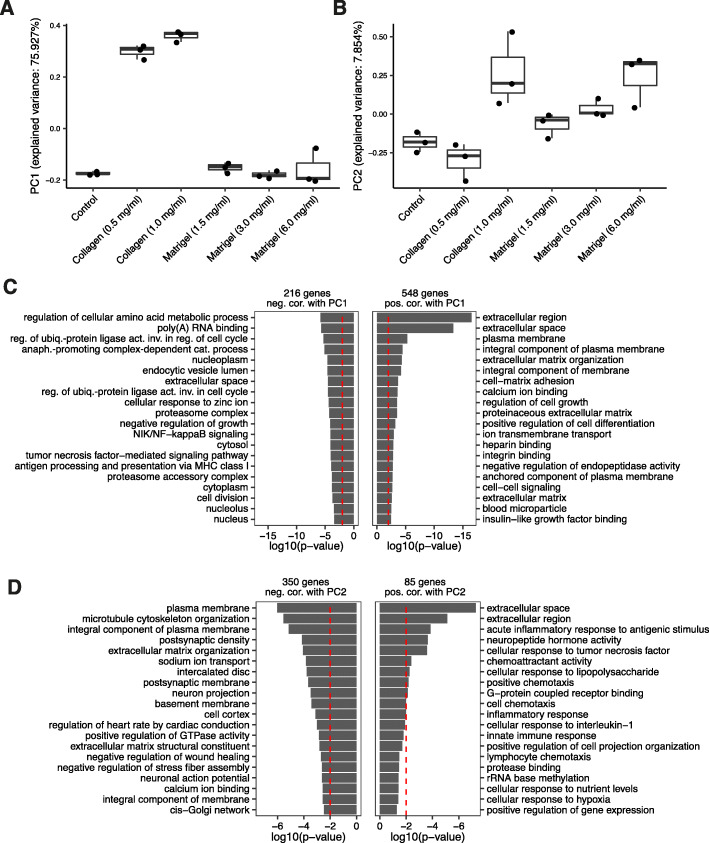
GO analysis of genes associated with the growth of Huh-7 on Matrigel and Collagen matrices. Principal component analysis (PCA) of the RNA-Seq data set prepared for samples of Huh-7 cells grown for 7 days on polystyrene coated cell culture plates (Control), or on Matrigel (1.5 mg/ml, 3.0 mg/ml or 6.0 mg/ml), or on Collagen (0.5 mg/ml and 1.0 mg/ml) basement membrane matrices. **a** Loadings of samples on PC1. **b** Loadings of samples on PC2. Each dot corresponds to one sample. Samples are grouped by conditions. The numbers associated with the principal components indicate the fraction of the variance in transcript expression that is explained by the corresponding principal component. **c** Gene ontology (GO) analysis of genes aligned with PC1. Positive and negative correlation coefficients are indicated as “neg. Cor.” or “pos. Cor.”, respectively. **d** GO analysis of genes aligned with PC2. GO analysis was performed with DAVID [16]. Numbers on the top of bar plots indicate the number of genes used for the GO analysis. Top 20 enriched GO terms were visualized. As a significance threshold for the enrichment, we considered *p*-value < 0.01 (dashed red lines)

To identify core transcriptional pathways that underlie the observed differences, RNA-seq samples from Huh-7 cells grown for 7 days on polystyrene-coated cell culture plates (Control), Matrigel (1.5 mg/ml and 3.0 mg/ml) or Collagen (0.5 mg/ml and 1.0 mg/ml) basement membrane matrices, were generated from three biological replicates (only two for Matrigel 1.5 mg/ml) per condition. The resulting RNA-Seq data set was subjected to principal component analysis (PCA) (Fig. 2a, b) and (Figure S1a, b, see Additional file 1). Strikingly, the first principal component (PC1), which explained about 75% of the variance present in the data set, distinguished cells cultured on the Collagen matrix from cells cultured in other conditions (Fig. 2a and Tables 1 and 2, see Additional file 2). In contrast, PC2 reflected the increase in concentration of matrix components (Fig. 2b and Tables 3 and 4, see Additional file 2). To identify the molecular changes that contributed most to the variance explained by PC1 and PC2, we aligned the vectors given by the gene expression levels of individual genes across all samples with PC1 and PC2. We further visualized the expression of genes whose expression levels were most aligned to PC1 and PC2 across all samples in a heat map (Figure S2a and Tables 1, 2, 3 and 4, see Additional files 1 and 2). This indicates that numerous genes are induced in the Collagen-based culture, while fewer have reduced expression in the same conditions. The gene ontology (GO) analysis with DAVID [16] revealed that genes aligned with PC1 corresponded to GO terms such as “extracellular region/space”, “calcium ion binding”, “regulation of cell growth”, “regulation of cellular amino acid metabolic process” and “proteasome complex” (Fig. 2c). Genes aligned with PC2 corresponded to the GO terms “extracellular region/space”, “inflammatory response” and “plasma membrane” (Fig. 2d). Note that although some of the GO terms that were enriched by genes aligned with PC1 and PC2 were similar, the enrichment was due to distinct sets of genes (Figures S3 and S4, see Additional file 1). Many genes involved in cell-cell adhesion and remodeling of cell-cell interactions (in the “extracellular region” category) had increased expression in the Collagen culture conditions (see Additional file 1). In contrast, genes involved in the “regulation of cellular amino acid metabolic process” and the “proteasome complex” had reduced expression in the Collagen culture condition. Consistent with the PC analysis, genes whose expression level changed significantly in Collagen compared to Control conditions were largely those that aligned well with PC1. For PC2, which reflected the concentration of the ECM components in the medium, only a limited number of genes were identified as differentially expressed in any one particular comparison of two conditions (Figure S2b, c, see Additional file 1).

**Fig. 3:**
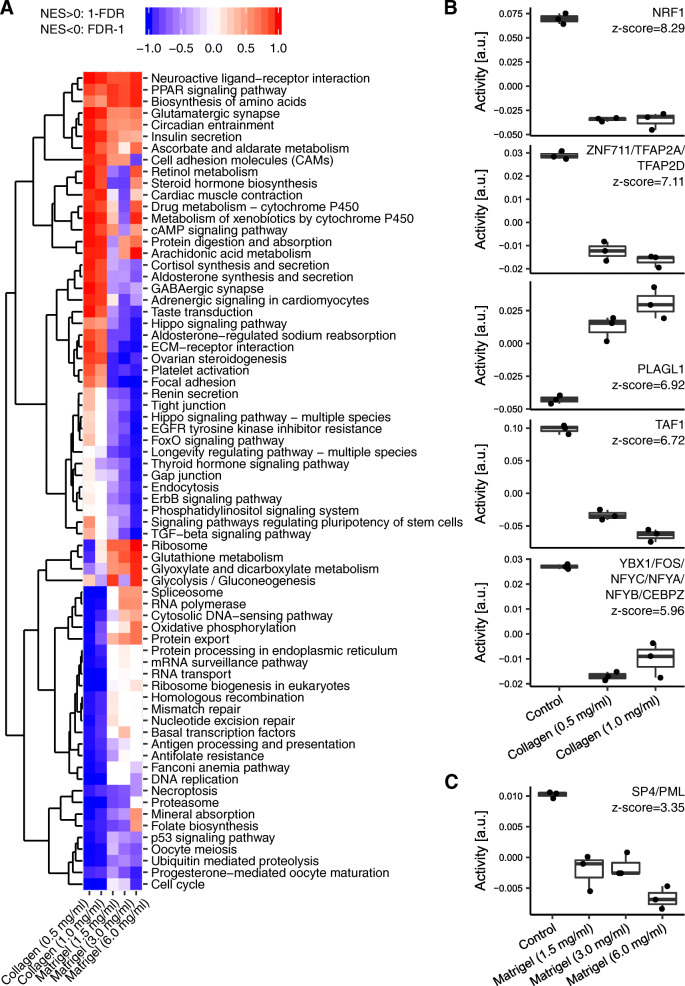
GSEA and Motif Activity Analysis. Pathway analysis in samples of Huh-7 cells grown for 7 days on polystyrene coated cell culture plates (Control), Matrigel (1.5 mg/ml, 3.0 mg/ml or 6.0 mg/ml) or Collagen (0.5 mg/ml and 1.0 mg/ml) basement membrane matrices. **a** Heatmap summarizing the enrichment of KEGG pathways among genes ranked by expression changes in cells grown on a basement membrane matrix in comparison to Control cultured cells. A pathway was included in the heatmap if it was significantly enriched for at least one condition. FDR < 0.05 was chosen as significance threshold. **b** Activities of the 5 most active motifs in Collagen culture condition. **c** Activity of the top motif in the Matrigel culture condition. Each dot corresponds to one sample. Samples are grouped by conditions. Z-scores indicate the contribution of regulatory motifs associated with TF(s) to explaining gene expression changes

To gain further insight into the remodelling of cellular activities depending on the culture conditions, we further carried out a gene set enrichment analysis (GSEA) [17] using KEGG pathways for functional annotation (Fig. 3a). This analysis revealed pathways that responded similarly to both ECM conditions, e.g., “Insulin secretion”, “Biosynthesis of amino acids” (both increasing), “p53 signaling pathway” and “Ubiquitin mediated proteolysis” (both decreasing). Other pathways responded in the opposite manner in cells grown on the Collagen matrix compared to cells grown on Matrigel, e.g., “ECM-receptor interaction”, “Focal adhesion” and “Cortisol synthesis and secretion”. Genes from pathways related to basic cellular functions such as “Ribosome”, “Spliceosome”, “Mismatch repair”, “mRNA surveillance pathway” and “RNA transport” were not affected or even somewhat increased by the culture in Matrigel, but were decreased in expression in the culture on Collagen. In contrast, pathways related to cell-cell adhesion and signalling, e.g. “Focal adhesion”, “Gap junction”, “FoxO signaling pathway” and “ErbB signaling pathway” were enhanced in the Collagen and decreased in the Matrigel condition.

The large numbers of genes and pathways that were regulated in Huh-7 cells upon changing the growth environment suggested the action of regulatory cascades involving specific transcription factors (TFs). To infer the TFs that may underlie the observed expression changes, we used the Integrated Motif Activity Response analysis tool (ISMARA [18]), which estimates activities of TFs based on transcriptome-wide RNA-seq data along with predictions of TF binding sites in promoters. Figure 3b depicts the inferred activities of the 5 most statistically significant binding motifs and associated TFs, from the comparison of Control cells with cells grown on the Collagen basement membrane matrix. Regulatory motifs whose activity decreased in cells grown on Collagen correspond to the TFs NRF1 (whose targets are annotated as “RNA polymerase”, “Chromosome organisation”), ZNF711/TFAP2A/TFAP2D (“Regulation of transferase activity”), TAF1 (“Spliceosome”, “RNA processing”) and YBX1/FOS/NFYC/NFYA/NFYB/CEBPZ (“Chromatin silencing”, “Protein folding” and “Cell cycle”). Targets of these TFs have reduced expression in the Collagen compared to Control condition, therefore the inference that these TFs have decreased activity. In contrast, the TF PLAGL1 (targets annotated as “Actin cytoskeleton”) had increased activity in cells grown on the Collagen basement membrane matrix. As the gene expression changes were milder in the Matrigel culture conditions, few TFs showed consistently changed activity in this condition. SP4/PML (targets annotated as “ECM”) was the TF that contributed most to the gene expression changes in Matrigel compared to Control culture conditions and showed consistency between replicates (Fig. 3c).

**Fig. 4:**
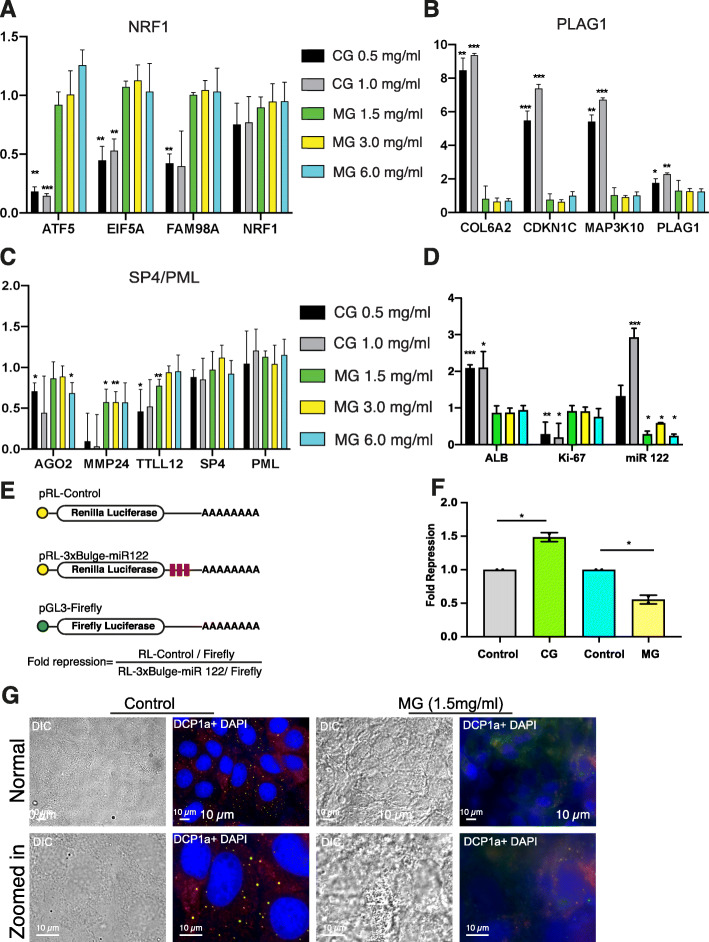
Relative expression levels of candidate genes linked to inferred TF activities. **a**-**c** Histograms representing the mean fold change of expression (+/− s.d.), estimated from normalised TPM (transcripts per million) counts, of the indicated genes in 3D culture normalised to their expression levels in Control 2D condition of growth. Results were obtained from three independent biological replicates (except Matrigel 1.5 mg/ml which had two replicates). Three top gene candidates of the statistically significant transcription factors are shown along with the expression level of the TF themselves for comparison. **d** Histograms represent the relative mean (+/− s.d.) expression levels of hepatocyte marker ALB, growth marker Ki-67 and mature miR-122-5p in cells grown on Collagen and Matrigel scaffolds. ALB, Ki-67 levels were estimated from normalised TPM counts. miR-122-5p levels were estimated by qPCR relative to U6snRNA levels. Histograms represent the enrichment in the quantities of the indicated genes in Collagen and Matrigl culture conditions normalised to their levels in the Control 2D growth condition. **e**-**f** Scheme of Plasmids used for transfection of Huh-7 cells and the basis of calculation of fold repression (**e**). Histograms represent relative repression levels of a miR-122 reporter with multiple bulged miR-122 binding sites in Huh-7 cells. Cells were co-transfected with Renilla / RL-3xBulge-miR 122 along with Firefly luciferase expression plasmid, split and re-seeded onto plates coated with Collagen (CG 0.5 mg/ml) or Matrigel (MG 1.5 mg/ml) or Control for a duration of 72 h prior to lysis and luminescence measurements. Experiments were performed with two biological replicates (with two technical replicates each) in separate batches for Control / MG and Control / CG. For comparison, the fold repression level was normalised to unit value in the Control condition (**f**). **g** Representative microscopic images of P bodies in Huh-7 cells grown on Control or Matrigel (1.5 mg/ml) reveals loss of discrete P bodies on Matrigel. For ectopic expression, a GFP tagged DCP1a expression construct was transfected into Huh-7 cells and subsequently the cells were split into Control or Matrigel coated chamber slides. Prior to imaging, Hoechst was added to stain the nucleus. A DIC image was also captured for visualisation of cell boundaries. A normal field of view image (top panel) and a digitally zoomed region (lower panel) of the field in the indicated conditions are shown. The *P* values of the unpaired non parametric t tests comparing the expression of the designated genes in the Matrigel and Collagen growth conditions, with respect to the Control, is denoted (when significant). The *P* values comparing the luciferase reporter repression levels, computed by a paired t test, is also denoted. * represents a *P* value of ≤0.05, ** represents a *P* value ≤0.01, *** represents a *P* value of ≤0.001. *P* values > 0.05 were considered non-significant

Mining the RNA-seq data, we verified that the top targets of the transcription factors mentioned above responded coherently to the culture conditions. We also examined the mRNA levels of the TFs themselves, which did not always change in correspondence to their activity (Fig. 4a-c). This is not entirely surprising, as the TF activity is frequently regulated by post-translational modifications [19]. Finally, we used the same data to evaluate the expression of some functional markers of hepatocytes. We found that relative to Control cells, the ALB (Albumin) hepatocyte marker was significantly upregulated in the cells grown on the Collagen matrix (Fig. 4d), consistent with the reported secretory activity of hepatocyte cells cultured in this condition [15]. In contrast, the growth marker Ki-67 was prominently reduced in Collagen, which is consistent with the downregulation of core cellular processes (Fig. 4d, and Fig. 3a). As an additional functional marker of hepatocytes, we estimated the levels of hsa-miR-122-5p, the microRNA which comprises more than 70% of the liver miRNA pool [20]. With around 66,000 copies of the miRNA per cell, miR-122 plays important roles in liver homeostasis, lipid metabolism [21] and also a very intriguing role in the hepatitis C virus (HCV) replication cycle [22]. Quantitative PCR revealed that miR-122 expression increased in the higher concentration of Collagen, while Matrigel led to a somewhat inconsistent downregulation relative to a housekeeping control U6 snRNA (Fig. 4d). In agreement with the altered miR-122 levels, we found that the levels of miR-122 reporter genes were proportionally altered on the ECM matrices (Fig. 4e, f). Hypothesizing that the ex-vivo consequence of the distinct reduction in miRNA activity would affect P bodies, the phase-separated compartments in the cells that are enriched in miRNA-repressed target mRNAs [23], we used fluorescently-tagged constructs expressing the P body marker DCP1a to document the reduction in discrete P body structures in the Matrigel condition (1.5 mg/ml) in comparison to the Control condition (Fig. 4g). Altogether, these data suggest that the Collagen culture accentuates some of the secretory and metabolic activities of hepatocytes, coordinated both by specific transcription factors and miRNAs. In contrast, the Matrigel condition induces milder gene expression changes and a distinct clustering of cells into spheroid like structures.

To further understand the impact of different culture conditions relative to the in vivo system of primary hepatocytes, we took advantage of a recently published data set [24] of RNA-seq reads from two primary hepatocyte isolations. Carrying out principal component analysis of our data along with the primary hepatocyte data, we found that PC1, which explains 77% of variance, distinguishes the gene expression profile of Huh7 cells from that of primary hepatocytes (Figure S5a and Table 5, see Additional file 2). In contrast, PC2 explains 16% of the variance and distinguishes the Collagen culture conditions from the Control and Matrigel culture conditions (Figure S5b and Table 6, see Additional file 2). The loadings of primary hepatocytes samples on PC2 are larger than those of Control and Matrigel-cultured Huh7 cell samples, but this difference was much smaller than that between Collagen and Control-cultured Huh7 cells. These results suggest that the concentration of Collagen that we used in our system strongly accentuates specific aspects of hepatocyte functionality.

**Fig. 5:**
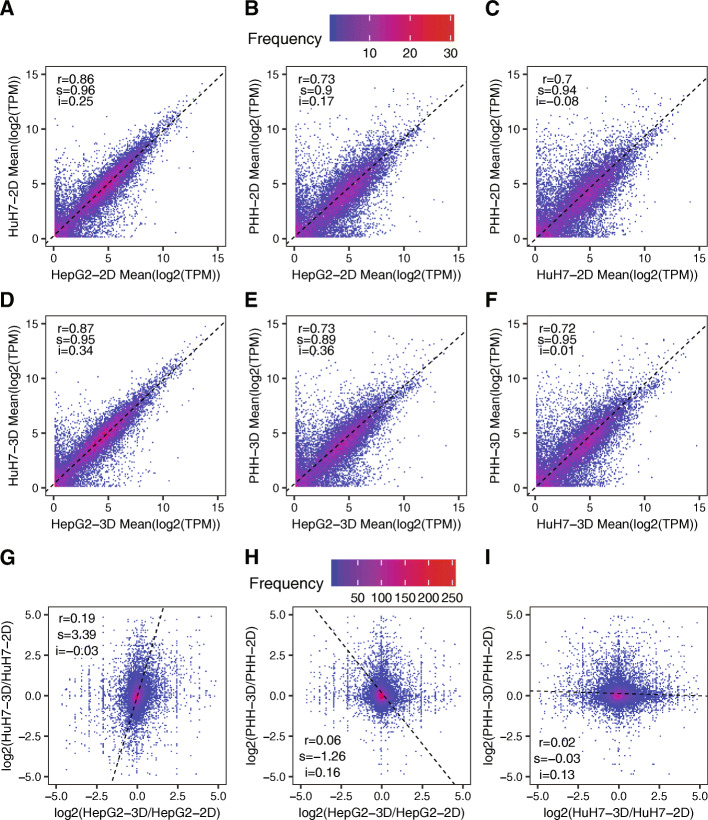
Correlation of the gene expression profiles of HuH7 cells, HepG2 cells and primary human hepatocytes (PHH) grown in 2D (standard culture for cell lines and Collagen/Matrigel ‘sandwich’ culture for PHH) and 3D (low-adherence plates to promote cell aggregation) conditions. **a**-**c** Pairwise correlation of the gene expression levels in different cell types grown in 2D culture. **d**-**f** Pairwise correlation of the gene expression levels in different cell types grown in 3D culture. The RNA-seq data came from the publicly available ERX2636883 record from the SRA database. ‘r’ indicates the value of the Pearson correlation coefficient. Black dashed lines correspond to directions of the highest variance (for comparison), with the slope ‘s’ and intercept ‘i’. **g**-**i** Correlation of gene expression changes in HuH7 cells, HepG2 cells and primary human hepatocytes (PHH) grown in 3D in comparison to 2D conditions. **g** HepG2 cells vs. HuH7 cells. **h** HepG2 cells vs. PHH cells. **i** HuH7 cells vs. PHH cells. The publicly available data set ERX2636883 from the SRA database was used for the analysis. ‘r’ indicates the value of the Pearson correlation coefficient. Black dashed lines correspond to directions of the highest variance for comparisons, with the slope ‘s’ and intercept ‘i’

To what extent do findings in cell lines generalize to primary cells remains a crucial question that is insufficiently addressed. Nevertheless, some data has started to emerge, particularly within large-scale projects that aim to establish standard data sets. An example is the Open Targets project of the European Bioinformatics Institute [25], which provides data and tools for drug target identification. Within this project, RNA-seq data (https://www.ncbi.nlm.nih.gov/sra/ERX2636883) has been obtained from both hepatic cell lines and primary hepatocytes, cultured either in 2D or 3D conditions. For cell lines, the 2D condition was similar to our Control, while for primary hepatocytes, the 3D condition used a sandwich configuration with two layers of Collagen coated plates and medium containing Matrigel for 9 days. The 3D condition involved ultra-low adherence multi-well plates that promoted cell aggregation. Analyzing the data for two hepatocyte cell lines (Huh-7 and Hep G2) and primary cells, we found that the cell line-derived were more similar to each other than either was to the data from primary cells, irrespective of the culture conditions (Fig. 5a-i). However, in spite of the reasonably high correlation within a condition, the change in gene expression levels between 3D and 2D conditions maintained a modest correlation for the cell lines, but no in the comparison of either cell line with those of primary cells (Fig. 5a-i). These results demonstrate that even within the same study, the generality of findings obtained in distinct cell systems is limited, further emphasizing the need to understand and optimize in vitro models.

## Discussion

Liver disease accounts for approximately 2 million deaths per year on a global scale. To alleviate the shortage of a reliable and readily available cell source, liver tissue engineering has emerged as a promising alternative approach in organ transplantation or for in vitro drug testing. Presently, the gold standard to test toxicity and metabolism consists of primary human hepatocyte cultures in 2D. However, 2D cultures fail to represent the complex 3D structure of native liver tissue [26]. Further problems associated with primary hepatocytes are limited availability, restricted lifespan, rapid dedifferentiation, functional variability, and a limit in drug transporter activity [27]. Moreover, despite their large replication capacity in vivo, hepatocytes remain difficult to expand in culture [28]. Thus, to be useful as a test bed for drug discovery, a cell line model needs to have a stable hepatic signature and the potential to expand.

Some of the salient features of hepatocytes that have been known for decades have also recently been shown to be non-uniformly represented among individual hepatocytes [29]. Specifically, this study identified six distinct sub-populations of hepatocytes, each liver zone having distinct clusters of cells with apparent functions ranging from gluconeogenesis/ β-oxidation to cholesterol and sterol biosynthesis. Some other clusters were characterised by the expression of amino acid metabolism genes or of genes involved in drug metabolism and detoxification. In this latter process the Cytochrome P450s (P450s) enzyme superfamily is responsible for the biotransformation of endogenous compounds, pharmacological agents, and environmental xenobiotics. P450 is a well-characterised liver marker whose variable activity can lead to severe pharmacological effects [30]. Due to a significant inter-species variability in the primary structure, function, and regulation of different P450 isoforms [31], it is difficult to extrapolate data between species, further underlining the need for in vitro hepatocyte models of human origin. Our transcriptomic data reveals that the expression levels of genes in this family can be tuned by the composition or the concentration of the ECM used for cell culture. Specifically, P450-related mRNAs are induced in Collagen culture conditions, while the induction is less robust in the Matrigel condition (Fig. 3a). In direct contrast to this pattern of expression, Gluconeogenesis was more prominently affected in Matrigel conditions with mild or almost unchanged patterns emerging from the culture of the cells on the Collagen matrix (Fig. 3a). As an essential metabolic organ, the metabolic activity of the liver is tightly controlled by hormones such as insulin and also by certain transcription factors [32]. Insulin-mediated alteration of FOXO-1 transcription factor activity has been extensively studied in the regulation of gluconeogenesis [33], where receptor-mediated signalling, through mTORC2-AKT, orchestrates the response of the liver to insulin levels in the body [34]. Transcripts linked to the oxidative phosphorylation were also upregulated on Matrigel, in agreement with the observed changes in the gluconeogenesis pathway (Fig. 3a). This is consistent with the findings that the hepatocytes can have very high mitochondrial content [35]. Yet another major role of the liver in human physiology is the biosynthesis of nonessential amino acids [36]. This pathway is ubiquitously upregulated in both ECM conditions, with a greater effect induced by Matrigel (Fig. 3a). Hepatocytes also function as a key node in the control of fatty acids and their neutral storage form which is the triglyceride [37]. A group of ligand-induced transcription factors that mediate transcription according to the fatty acid nutritional state is the peroxisome proliferator-activated receptor (PPAR) family [38]. Comprising different isoforms, the PPARα is predominantly expressed in hepatocytes where they are involved in fatty acid oxidation [39]. A careful percipience of our data also reveals a heightened PPAR signalling response in the cultures on ECM components, indicating an enhanced hepatocyte functionality of these cells compared to those cultured in Control conditions (Fig. 3a).

As a readout of the remodeled hepatocytes, we found evidence of the alterations in the levels of the ALB gene and also of the mature miRNA 122 (Fig. 4d). Several other clusters of gene changes observed in our data sets need to be critically scrutinised for further understanding and better manipulation of hepatocyte functions and to aid development of alternate scaffold material. Taking note of the apparent feedback regulation of the ECM-Receptor interactions (Fig. 3a) and the visible changes in the cellular structure observed under the phase contrast microscope (Fig. 1), it is likely that the concentrations of each component of the scaffold matrix need to be further optimised and fine-tuned to achieve a cellular state akin to a hepatocyte. In parallel, a quantitative nanomechanical profiling of the cells with atomic force microscopy would need to be performed to better link the ECM stiffness to the mechano-cellular phenotype changes, at the level of the cytoskeleton and focal adhesions [40].

Our observations also consolidate earlier reports that have indicated a need to use caution in interpreting results based on Matrigel-cultured cells [41, 42] which has been the gold standard for several 3D studies. In itself, Matrigel has several fundamental drawbacks related to the variability in its composition and the presence of xenogenic contaminants even within single batches. In comparison, Collagen has been reported to impede invasive growth by providing a matrix metalloprotease barrier (MMP barrier) to the cell, which is not present in Matrigel [43]. This may partly explain the visibly different growth pattern in Matrigel, although the transcriptional response is not as substantial as cells grown on Collagen. Analyses of the transcriptional networks that are triggered in different culture conditions could elucidate the molecular underpinnings of these different growth patterns.

Emerging trends in the fields of iPSC derived / Embryonic stem cell derived hepatocytes [44–46] are very encouraging but at this time, there are limitations, including the cost and time needed to maintain and differentiate cells into hepatocytes, as well as the potential genetic instability associated with the reprogramming strategy. These pitfalls need to be considered with regard to clinical applications and therefore it is more prudent (and frugal), in the current scenario, to remodel hepatocyte cell lines to hepatocytes, particularly for use in research.

## Conclusion

Our study reveals large differences in gene expression attributable to the specific culture conditions of Huh-7 and it highlights transcription factors that seem to underlie these differences. Furthermore, we observe that Collagen matrix scaffolds provide an improved and consistent remodelling of hepatoma cell lines towards primary hepatocytes. Our analysis also highlights the importance of the concentration and composition of the scaffold material in triggering a feedback response on the transcriptome. We believe that our robust characterization of the gene expression in hepatocytes will spur the design of future mechanistic studies, exploiting transcription factors as hubs for global changes, geared towards the development of a readily available and sustainable in vivo model for the study of liver function and disease.

## Methods

### Coating of plates with collagen and Matrigel matrices

#### Collagen coating

Collagen coating of plates was performed inside a cell culture hood. Initially, Collagen (Thermo Fisher Scientific, Catalogue #A1048301), sterile 10X phosphate buffered saline (Thermo Fisher Scientific, Catalogue #AM9625), sterile distilled water (dH2O), and sterile 1 N NaOH (Merck, Catalogue # S2770-100ML) was thawed on ice. For our experiment we used final Collagen concentration of 1 mg/ml and 0.5 mg/ml. Total volume of collagen stock required for preparation of each dilution was calculated as per manufacturers protocol (Thermo Fisher Scientific, Catalogue #A1048301)

Subsequently, in a sterile tube, dH2O, 1 N NaOH, and 10X PBS were mixed. The estimated amount of Collagen was slowly pipetted into the tube, with multiple rounds of pipetting the solution up and down to achieve a homogeneously mixed solution. The resulting mixture was checked to ensure that it achieved a pH of 6.5–7.5 (optimal pH is 7.0). The diluted Collagen solution was then dispensed carefully into the plates (500 μl per well of a standard 6 well tissue culture plates), on ice. The ice was used to ensure uniform coating of the plate with the cold temperature thwarting rapid gelling at room temperature. The plates were then incubated at 37 °C in a humidified incubator for 30–40 min until a firm gel was visually observed. Lastly, the wells were rinsed twice with 1X PBS and cell culture medium before seeding of cells.

#### Matrigel® growth factor reduced (GFR) basement membrane matrix coating

To prepare tissue culture plates for Matrigel (Corning, Catalogue # 354230) coating, which would be used for seeding cells, the Matrigel matrix was thawed before use overnight by submerging the vial in a 4 °C refrigerator. Once the Matrigel matrix was thawed, the vial was swirled several times on ice to ensure even dispersion of material. We opted for three different concentrations of Matrigel (1.5 mg/ml, 3.0 mg/ml and 6.0 mg/ml) for coating the cell culture plates. The stock Matrigel solution was diluted with ice cold Opti-MEM I Reduced Serum Media (Thermo Fisher Scientific, Catalogue # 11058021). The solutions were thoroughly mixed and then kept on ice until further use. Additionally, cell culture plates, tip boxes to be used were also pre-conditioned in a 4 °C refrigerator before use. The diluted Matrigel solutions (500 μl) prepared earlier were dispensed into each well of a 6 well plate in triplicates for each dilution. The culture plates were transferred to a 37 °C humidified incubator for gelling of the Matrigel solutions for at least 30 min or until visible gelling was observed. The wells were rinsed with 1X PBS and cell culture medium before seeding of cells.

### Cell culture and imaging

Huh-7 cells grown in cell culture flasks were washed with PBS, and then trypsinized with Trypsin-EDTA (0.05%), phenol red (Thermo Fisher Scientific, Catalogue # 25300054). Following completion of cell detachment from the flask, growth media (DMEM high glucose (Sigma, Catalogue #D6546) supplemented with 2 mM L-Glutamine (ThermoFisher Scientific, Catalogue #25030081) and 10% Fetal Calf Serum (FCS) (Amimed, Catalogue #2-01F00-I) was added to stop the process. Subsequently the cells were spun in a centrifuge at 200 g in a swinging bucket rotor and washed twice with PBS. Finally the cell pellet was suspended in growth media at a concentration of 5000 cells/μl. 25,000 cells were then used to seed each well of a 6 well plate with or without the basement matrices as prepared earlier. Cells were observed every day, with growth media being changed every alternate day for a total duration of a week. At 1 day and 7 days post seeding, cells were imaged with an Olympus Phase Contrast microscope, and live snapshots were taken with an Olympus CKX41 inverted microscope equipped with a SC 30 digital camera (Olympus). The images were procured on an inverted phase contrast microscope with Cell Sens software. All images documented were then processed with OMERO (an initiative of the open microscopy environment https://www.openmicroscopy.org/) for digital zoom.

### RNA-Seq

#### Sample preparation and sequencing

Total RNA from the three biological replicates of each sample (except for Matrigel 1.5 mg/ml, for which only two libraries were used) was quality-checked on the Bioanalyzer instrument (Agilent Technologies, Santa Clara, CA, USA) using the RNA 6000 Nano Chip (Agilent, Cat# 5067–1511), quantified by Spectrophotometry using the NanoDrop ND-1000 Instrument (NanoDrop Technologies, Wilmington, DE, USA) and adjusted to a concentration of 20 ng/μl. 1 μg total RNA was used for library preparation with the TruSeq Stranded mRNA Library Prep Kit High Throughput (Cat# RS-122-2103, Illumina, San Diego, CA, USA). Libraries were quality-checked on the Fragment Analyzer (Advanced Analytical, Ames, IA, USA) using the Standard Sensitivity NGS Fragment Analysis Kit (Cat# DNF-473, Advanced Analytical). The average size of the fragments was ~ 250 bases. Samples were pooled to equal molarity. Each pool was quantified by PicoGreen Fluorometric measurement to be adjusted to 1.8pM and used for clustering on the NextSeq 500 instrument (Illumina). Samples were sequenced (single end: read length 76) using the NextSeq 500 High Output Kit 75-cycles (Illumina, Cat# FC-404-1005). Primary data analysis was performed with the Illumina RTA version 2.4.11 and base calling software version bcl2fastq-2.20.0.422.

#### RNA-Seq data processing

RNA-Seq reads were subjected to 3′ adapter (5′-AGATCGGAAGAGCACACGTC-3′) and poly(T) trimming at the 5′ end using Cutadapt v1.9.1 [47]. Trimming of poly(T) stretches at the 5′ end was performed, because the RNA-seq reads originated from the antisense strand. Reads shorter than 30 nucleotides were discarded. As the reference human transcriptome, we considered sequences of protein-coding transcripts with support level 1–3 based on the genome assembly version GRCh38 (release 96) and the transcript annotation from Ensembl database [48]. The kallisto v0.43.1 software was used to assign the filtered reads to transcripts [49]. The default options of kallisto were utilized for building the transcriptome index. For aligning single-end RNA-Seq reads we used the options “--single” and “-l” and “-s”, the latter corresponding to the mean and standard deviation of fragment length, respectively, estimated for each sample with the BioAnalyzer instrument. Since reads originated from the antisense strand, the option “--rf-stranded” was used. The option “--pseudobam” was used to save kallisto pseudoalignments to a BAM file.

Mapped reads were then assigned to transcripts in a weighted manner: if a read was uniquely mapped to a transcript, then the transcript’s read count was incremented by 1; if a read was mapped to *n* different transcripts, each transcript’s read count was incremented by 1/*n*. Trimming the 3′ adapter and poly(T) stretches, indexing the reference transcriptome, mapping the RNA-Seq reads to transcripts and counting the reads assigned to individual transcripts were performed with a Snakemake framework [50].

The expression of each transcript *t*_*i*_ was then estimated in units of transcripts per million (TPM) by dividing the read count *c*_*i*_ corresponding to the transcript by the transcript length *l*_*i*_ and normalizing to the library size:

$$ {t}_i=\frac{\frac{c_i}{l_i}}{\sum_{j=1}^{\# of\ transcripts}\frac{c_j}{l_j}}\cdotp {10}^6. $$

The expression level of a gene was calculated as the sum of normalized expression levels of the transcripts associated with the gene.

Public data sets were processed according to the method described above. The data of [24] was single end and the only modification was to assume a mean and s.d. of fragment length of 300 and 100, respectively, as this information was not provided in the record. The data from the Open Targets project (https://www.ncbi.nlm.nih.gov/sra/ERX2636883) was paired-end, and for these, the fragment length could be inferred from the data themselves. The adapter trimmed for the Read2 was 5′-AGATCGGAAGAGCGTCGTGT-3′. The adapter for Read1 was the same as for other data sets.

#### Aligning gene expression with principal components

The gene expression matrix (log2-transformed normalized gene expression values in TPM units) with samples as columns and genes as rows was centered to make the data comparable both across samples and genes. The centered gene expression matrix was further subjected to the principal component analysis (PCA). For further analyses, we focused on the first two principal components, PC1 and PC2, which explained ~ 85% of the variance (Fig. 2 a, b).

We quantified how much individual genes contributed to PC1 and PC2, respectively, and identified genes that induced the difference between samples indicated by these PCs [51, 52]. The gene expression values were randomly sampled from the multivariate normal distribution. Here, every gene can be represented as a vector in the sample space, where rows of the centered gene expression matrix correspond to the coordinates of these vectors. Black vectors indicate PC1 and PC2, a representative gene is indicated by a blue vector. Figure S1 (see Additional file 1) illustrates this concept of the analysis on a simulated data set consisting of three ‘samples’ and 26 ‘genes’ measured for each sample. For determining the contribution of individual genes to a PC, we calculated both the magnitude of the projection of gene vectors on the principal component (PC) (dashed blue line) and the correlation with the PC (cos *α*). Genes for which the absolute correlation was ≥ 0.6 and the absolute z-score of the projection was ≥ 1.96 were extracted as contributing most to the variance explained by the PC.

#### Gene ontology analysis

To characterize functions of genes contributing most to PC1 and PC2, we performed gene ontology (GO) analysis using the Database for Annotation, Visualization and Integrated Discovery (DAVID) [16] tool and the “GOTERM_BP_DIRECT”, “GOTERM_MF_DIRECT” and “GOTERM_CC_DIRECT” categories. The reference gene list consisted of those genes that had an expression value of at least 1 TPM in a number of samples corresponding to the minimum number of samples in each condition. We considered a GO term to be significantly enriched if the corresponding *p*-value was smaller than 0.01. For inspecting protein-protein interactions of genes from top enriched GO terms we used STRING (https://string-db.org/) [53].

#### Differential expression analysis

Differential expression analysis was performed with EdgeR, available through the Bioconductor package [54]. Note that a gene was included in the analysis only if it had at least 1 count per million (CPM) in the number of samples corresponding to the minimum number of samples in each condition. Using EdgeR, log2-fold changes in the gene expression in Huh-7 cells grown on basement membrane matrices in comparison to cells grown on Control polystyrene coated cells were calculated and further used for the gene set enrichment analysis. These read counts were used for calculating differential gene expression levels as shown in Fig. 4.

#### Gene set enrichment analysis

The distribution of genes from KEGG pathways (http://www.kegg.jp) [55] in ranked gene lists was examined using gene set enrichment analysis (GSEA) [17]. Ranked gene lists were formed based on log2-fold changes in the gene expression of Huh-7 cells cultured on a given basement membrane matrix in comparison to Control polystyrene coated cell culture plates. The enrichment was considered to be significant if the corresponding false discovery rate (FDR) was smaller than 0.05. For visualizing GSEA results we considered both the FDR and the normalized enrichment score (NES) indicating if the enrichment was based on genes that were up-regulated (in which case NES > 0 and we used 1-FDR as significance measure for the visualization) or down-regulated (in which case NES < 0 and we used FDR-1 for visualization) (Fig. 3a). The calculated significance measures were further used for the hierarchical clustering, which was done based on the Euclidean distance.

#### Estimation of transcription factor activities

We used ISMARA [18] to estimate the activity of all transcription factors (TFs) with known binding specificity in both Control and ECM-based Huh-7 cell cultures. In brief, ISMARA models the expression levels of all mRNAs in a sample in terms of the activity of all TFs as well as the response of respective gene promoters. The response of a promoter to a TF is assumed to be the number of predicted binding sites the promoter has for the respective TF. The explanatory power of any given regulatory motif in the data set is then represented by its z-score.

### qRT-PCR for miRNA 122

Real-time analyses by two-step RT–PCR were carried out to quantify miRNA expression using the Thermo Scientific TaqMan chemistry-based miRNA assay system as performed earlier [56]. 25 ng of cellular RNA were used along with specific primers for human miR-122 (assay ID 000445). U6 snRNA (assay ID 001973) was used as an endogenous Control. One third of the reverse transcription mix was subjected to PCR amplification with TaqMan® Universal PCR Master Mix No AmpErase (Thermo Scientific) and the respective TaqMan® reagents for target miRNA. Samples were analyzed in PCR triplicates from at least two biological replicates. The comparative Ct method which included normalization by the U6 snRNA, was used for each cell type for plotting of mean values with s.d.

### Fluorescence microscopy

GFP-DCP1a, cloned within NcoI and XhoI sites of peGFP-C2 plasmid of Clontech [57], was used for transfection of Huh7 cells. For imaging, cells were transfected with 500 ng of GFP-DCP1a plasmid in a well of a 12 well plate. The cells were split after 24 h of transfection and subjected to specific experimental conditions like Control and Matrigel (1.5 mg/ml) with a very thin coating, to avoid imaging bottlenecks. The cells were then allowed to grow for 72 h before being imaged with an imaging system with a Plan Fluor 10X/0.30 objective on an inverted Eclipse Ti Nikon microscope equipped with a QImaging-Rolera EMC2 camera for image capture. Nucleus was stained with Hoechst 33342 (ThermoFisher Scientific) as per manufacturer’s protocol. Direct visualisation of fluorescent signals was made from cells in their native growth condition without fixation in 3D followed by deconvolution with software from Nikon.

### Luciferase assay

pRL-Con contains the humanized Renilla Luciferase coding region. pRL-3xBulge-miR122 contains three miR-122 binding sites downstream of Renilla Luciferase (RL) coding region. pGL3FF (Promega) encodes a Firefly Luciferase (FL) gene under the SV40 promoter. The RL-Con/RL-3xBulge-miR122 was cotransfected with FL plasmids in 6-well plates, as described earlier [58]. The cells were then split after 24 h into plates with thin coatings of Collagen and Matrigel (with the lowest concentrations to avoid cell lysis problems) or into Control plates. The cells were then allowed to grow for 72 h before the relative activities were measured using a Dual-Luciferase Assay Kit (Promega, Madison, WI) following the supplier’s protocol on a Mithras Luminometer (Berthold Technologies). Two independent experiments with two technical replicates were assayed for each condition (with individual Controls).

## Supplementary Information

**Additional file 1.**

**Additional file 2.**

## Data Availability

The RNA-seq data are available in the GEO database [59, 60] as “The transcriptional landscapes of a hepatoma cell line grown on scaffolds of Extracellular Matrix proteins” , accession GSE142206.The code for the analysis of RNA-Seq data presented in the manuscript is available on GitHub: https://github.com/zavolanlab/Liver3DCulture.
