# Supplementary material for: The transcriptional landscape of a hepatoma cell line grown on scaffolds of extracellular matrix proteins
Source: BMC Genomics. 2021 Apr 6;22:238. doi: 10.1186/s12864-021-07532-2 (PMC8025518; doi:10.1186/s12864-021-07532-2)
Supplement: Supplementary file 1 — Additional file 1. [file 12864_2021_7532_MOESM1_ESM.pdf]

Additional File 1 for

"The transcriptional landscape of a hepatoma cell line grown on scaffolds of extracellular matrix proteins"

Authors

Souvik Ghosh[1,2,3,4], Anastasiya Börsch[1,2,3] , Shreemoyee Ghosh[1] and Mihaela Zavolan[1,2,4]

Affiliations

1. Biozentrum, University of Basel, Switzerland

2. Swiss Institute of Bioinformatics, Switzerland

3. Equal Contribution

4. Corresponding author(s): Souvik Ghosh (souvik1983@gmail.com), Mihaela Zavolan (mihaela.zavolan@unibas.ch)

A

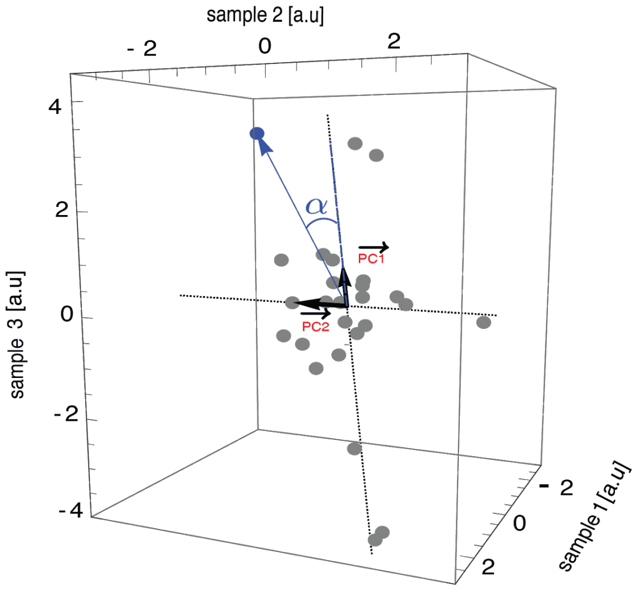

B

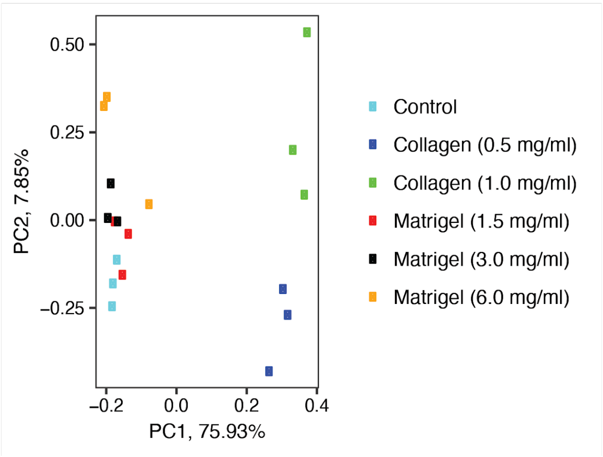

Figure S1. **Aligning gene expression with principal components.** (A) Visualization of the projection (blue dashed line) of a representative gene vector (blue arrow) on PC1 (black arrow) . The corresponding angle between the gene vector and PC1 is marked as α. The correlation between the gene vector and PC1 corresponds to cosα. “a.u” represents arbitrary units. (B) Principal component analysis (PCA) of the RNA-Seq data set prepared for samples of Huh-7 cells grown for 7 days on polystyrene coated cell culture plates (Control), or on Matrigel (1.5 mg/ml and 3.0 mg/ml), or on Collagen (0.5 mg/ml and 1.0 mg/ml) basement membrane matrices. Each dot

corresponds to one sample with colors indicating the culture condition. The numbers associated with PCs indicate the fraction of the variance in gene expression across samples along the corresponding PC.

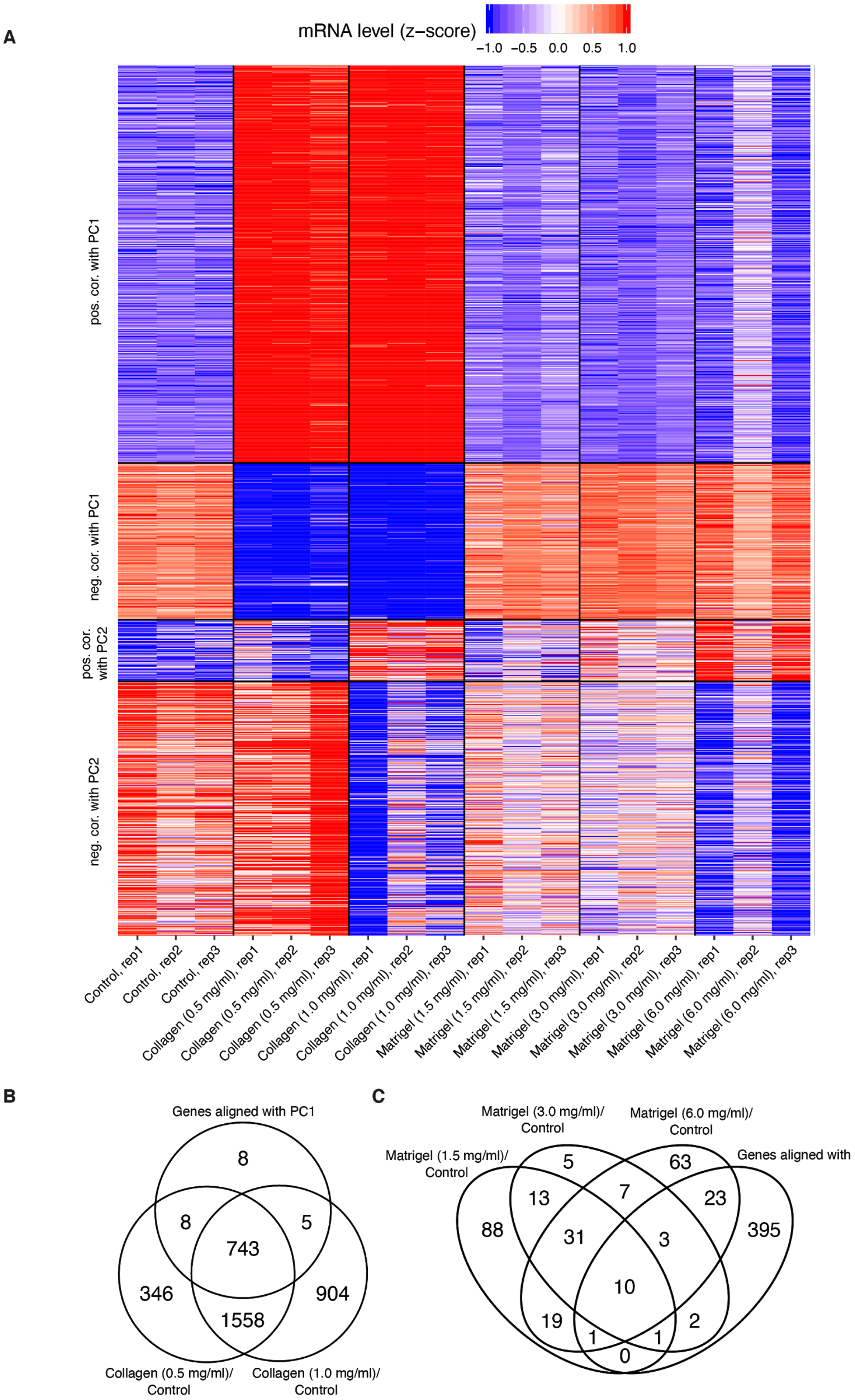

A
mRNA level (z−score)
−1.0
−0.5
0.0
0.5
1.0
pos. cor. with PC1
neg. cor. with PC1
pos. cor. with PC2
neg. cor. with PC2
Control, rep1
Control, rep2
Control, rep3
Collagen (0.5 mg/ml), rep1
Collagen (0.5 mg/ml), rep2
Collagen (0.5 mg/ml), rep3
Collagen (1.0 mg/ml), rep1
Collagen (1.0 mg/ml), rep2
Collagen (1.0 mg/ml), rep3
Matrigel (1.5 mg/ml), rep1
Matrigel (1.5 mg/ml), rep2
Matrigel (1.5 mg/ml), rep3
Matrigel (3.0 mg/ml), rep1
Matrigel (3.0 mg/ml), rep2
Matrigel (3.0 mg/ml), rep3
Matrigel (6.0 mg/ml), rep1
Matrigel (6.0 mg/ml), rep2
Matrigel (6.0 mg/ml), rep3
B
Genes aligned with PC1
8
8
743
5
346
1558
904
Collagen (0.5 mg/ml)/ Control
Collagen (1.0 mg/ml)/ Control
C
Matrigel (3.0 mg/ml)/ Control
Matrigel (6.0 mg/ml)/ Control
Matrigel (1.5 mg/ml)/ Control
Genes aligned with
5
63
13
7
23
88
31
3
395
10
19
2
1
1
0

Figure S2. **Heatmap analysis.** (A) Expression of genes aligned with PC1 and PC2 and contributing most to the variance in the RNA-Seq data that is explained by these PCs (Also see additional file 2). (B-C) Venn diagram represents the intersection of the genes aligning to PC1 (B) and to PC2 (C) in the various experimental conditions tested in the study.

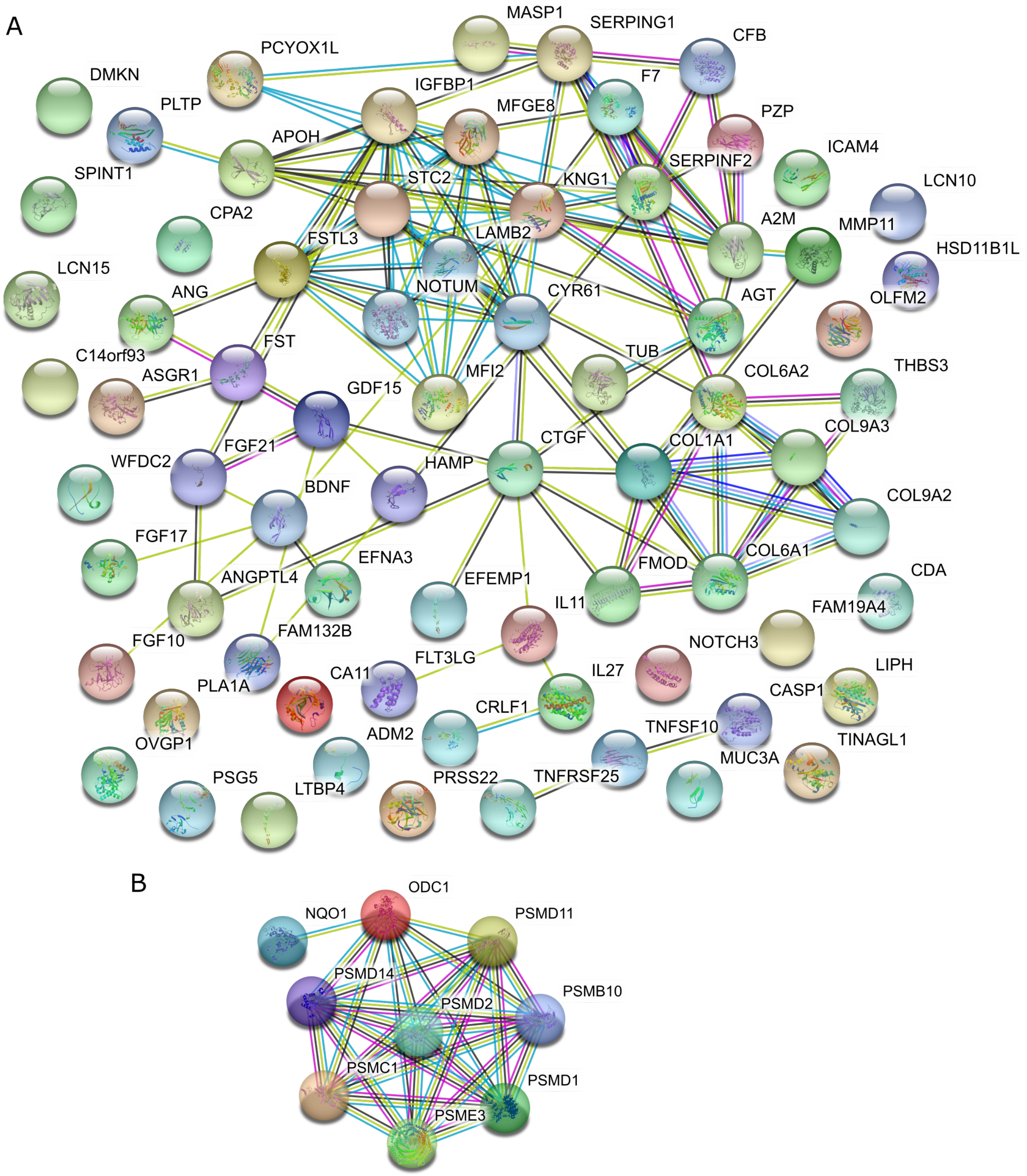

Figure S3. Gene network analysis. STRING networks of genes aligned with PC1 and associated with the top enriched GO categories. (A) Genes associated with the GO term "Extracellular

region”. (B) Genes associated with the GO term “Regulation of cellular amino acid metabolic process”.

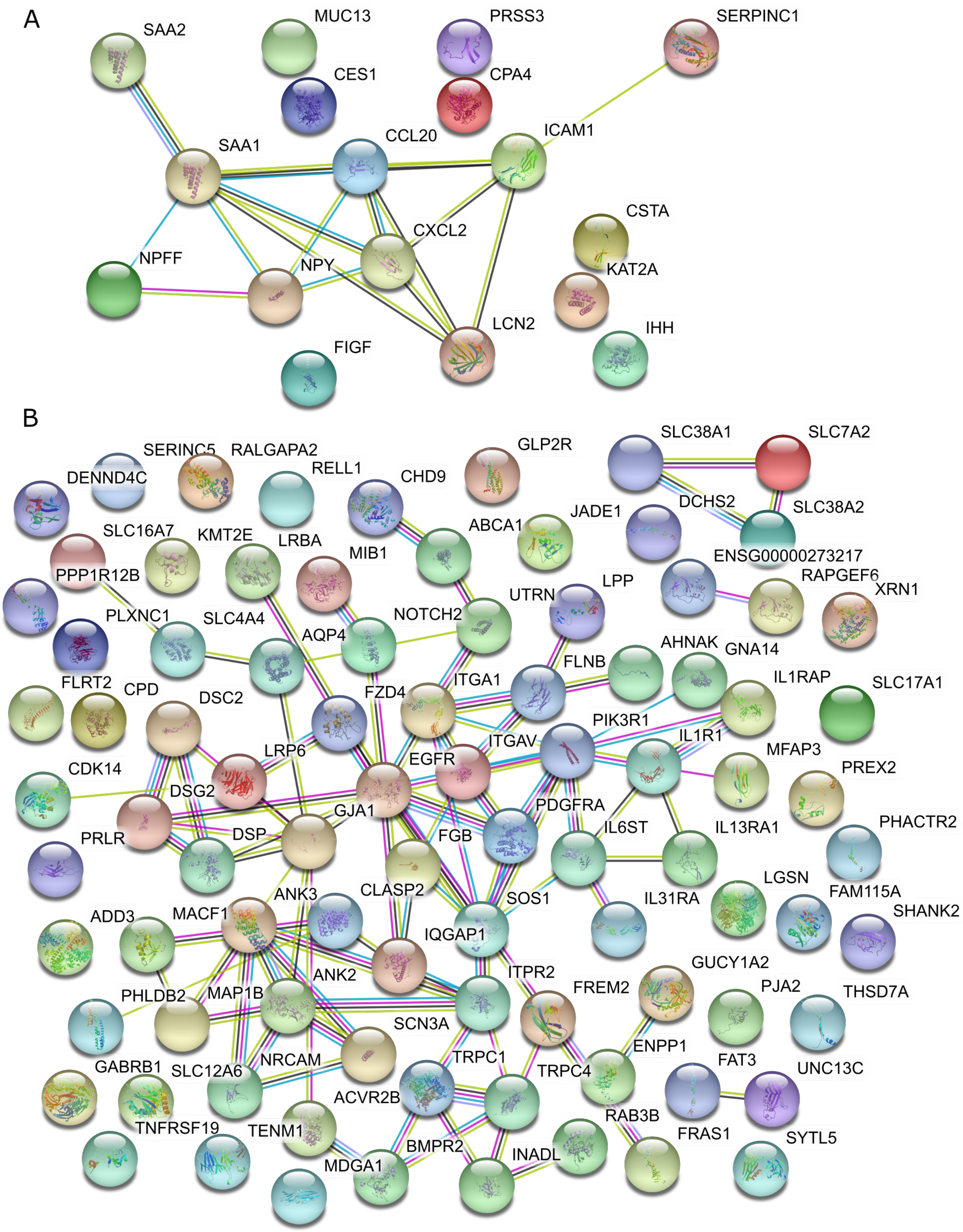
A
SAA2
MUC13
PRSS3
SERPINC1
CES1
CPA4
SAA1
CCL20
ICAM1
CSTA
CXCL2
NPFF
NPY
KAT2A
LCN2
IHH
FIGF
B
SERINC5
RALGAPA2
GLP2R
SLC38A1
SLC7A2
DENND4C
RELL1
CHD9
DCHS2
SLC38A2
SLC16A7
KMT2E
LRBA
ABCA1
JADE1
ENSG00000273217
PPP1R12B
MIB1
UTRN
LPP
RAPGEF6
XRN1
PLXNC1
SLC4A4
AQP4
NOTCH2
AHNAK
GNA14
FLNB
FLRT2
CPD
DSC2
FZD4
ITGA1
IL1RAP
SLC17A1
PIK3R1
ITGAV
LRP6
EGFR
IL1R1
MFAP3
CDK14
DSG2
GJA1
PREX2
PDGFRA
IL6ST
IL13RA1
PHACTR2
PRLR
DSP
FGB
LGSN
FAM115A
ANK3
CLASP2
SOS1
IL31RA
SHANK2
ADD3
MACF1
IQGAP1
GUCY1A2
ITPR2
PJA2
THSD7A
ANK2
FREM2
PHLDB2
MAP1B
SCN3A
ENPP1
FAT3
UNC13C
GABRB1
SLC12A6
NRCAM
TRPC1
TRPC4
ACVR2B
RAB3B
TNFRSF19
TENM1
FRAS1
SYTL5
BMPR2
INADL
MDGA1

Figure S4. STRING networks of genes aligned with PC2 and associated with the top enriched GO categories. (A) Genes associated with the GO term "Extracellular space". (B) Genes associated with the GO term "Plasma membrane".

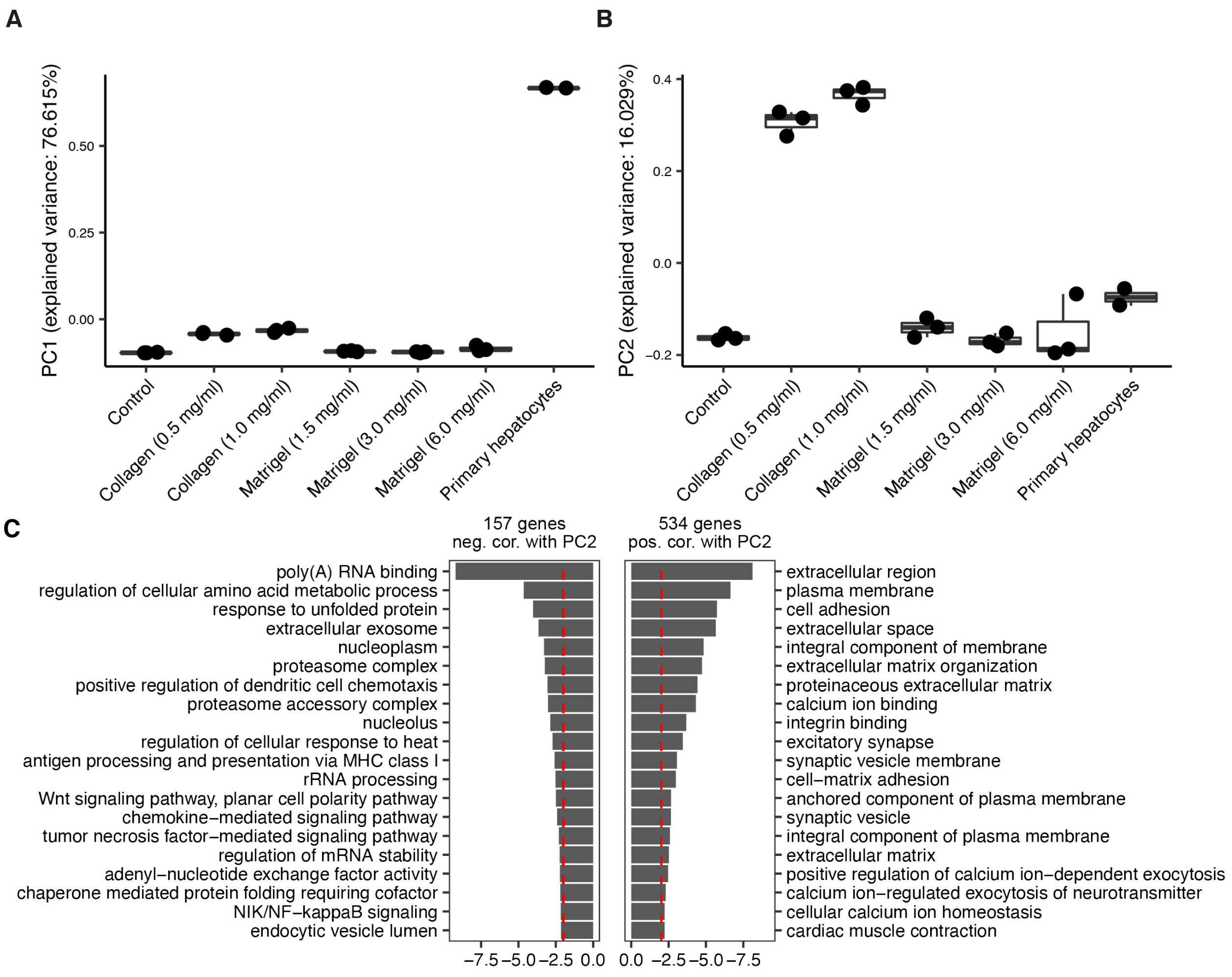

Figure S5. Principal component analysis (PCA) of the RNA-Seq data set generated in this study (prepared from samples of Huh-7 cells grown for 7 days on polystyrene coated cell culture plates (Control), on Matrigel (1.5 mg/ml and 3.0 mg/ml), or on Collagen (0.5 mg/ml and 1.0 mg/ml) basement membrane matrices), along with the data obtained from freshly isolated primary human hepatocytes [1]. (A) Loadings of the samples on PC1. (B) Loadings of the samples on PC2. Each dot corresponds to one sample. Samples are grouped by conditions. The numbers associated with the principal components indicate the fraction of the variance in transcript expression that is explained by the corresponding principal component. (C) Gene ontology (GO) analysis of genes aligned with PC2. GO analysis was performed with DAVID [2]. Numbers on the top of bar plots indicate the number of genes used for the GO analysis. Top 20 enriched GO terms were visualized. As a significance threshold for the enrichment we considered p-value < 0.01 (dashed red lines). 'neg. cor' and 'pos. cor' designate 'negatively correlated' and 'positively correlated', respectively.

References

1. Schneeberger K, Sánchez-Romero N, Ye S, van Steenbeek FG, Oosterhoff LA, Pla Palacin I, et al. Large-Scale Production of LGR5-Positive Bipotential Human Liver Stem Cells. Hepatology. 2020;72:257–70.

2. Huang DW, Sherman BT, Lempicki RA. Systematic and integrative analysis of large gene lists using DAVID bioinformatics resources. Nature Protocols. 2009;4:44–57. doi:10.1038/nprot.2008.211.
